# The success of Eso-SPONGE® therapy in the treatment of anastomotic dehiscence after Ivor-Lewis subtotal esophagectomy: A case report

**DOI:** 10.1016/j.ijscr.2021.106525

**Published:** 2021-10-18

**Authors:** Lorenzo Federico Zini Radaelli, Beatrice Aramini, Angelo Ciarrocchi, Stefano Sanna, Desideria Argnani, Franco Stella

**Affiliations:** Thoracic Surgery Unit, Dipartment of Diagnostic and Specialty Medicine - DIMES of the Alma Mater Studiorum, University of Bologna, G.B. Morgagni - L. Pierantoni Hospital, 34 Carlo Forlanini Street, 47121 Forlì, Italy

**Keywords:** Eso-SPONGE®, Esophageal dehiscence case report, Surgery of esophagus, Esophagectomy, Esophageal anastomosis, Ivor-Lewis's technique

## Abstract

**Introduction:**

Eso-SPONGE® has proved to be an excellent method for the treatment of persistent dehiscence of the intrathoracic esophagogastric anastomosis during the operation of subtotal esophagectomy *sec*. Ivor Lewis.

**Clinical case presentation:**

The case presented is of a 72-year-old patient with esophageal adenocarcinoma (ADK) who underwent sub-total esophagectomy and esophagoplasty sec. Ivor Lewis complicated by an esophageal leak. The Eso-SPONGE® therapy has been successful halving the index of inflammation after the first two sessions and generation of a neowall after seven sessions.

**Discussion:**

Eso-SPONGE® therapy has proven to be a valuable resource as a treatment for esophageal anastomotic dehiscences because it is easily repeatable in suburban centers, provided that they have a digestive endoscopy specialized in the positioning process.

**Conclusions:**

Eso-SPONGE® is a minimally invasive method that delivers excellent results in the treatment of fragile patients, such as those who have post-esophageal anastomotic dehiscence.

## Highlights

• Anastomotic dehiscence is an adverse event in esophageal surgery.
• Eso-SPONGE® is an excellent method to treat the esophageal post-surgery dehiscence.
• Eso-SPONGE® has provided better results than previous methods.
• Eso-SPONGE® is easily repeatable even in not-expert centers.

## Abbreviations

BMI: Body max index
C: contrast
HRCT: high resolution computed tomography
EGDS: esophagogastroduodenoscopy

## 1 Introduction and importance

In the surgery of sub-total esophageal and plastic esophagus *sec*. Ivor-Lewis, the most feared complication is anastomotic dehiscence, which puts the life of the patient at serious risk with a mortality of about 20% [1], [2]. Anastomotic dehiscence is an adverse event that occurs in 5–30% of treated patients, and its onset has a multifactorial etiology [3], [4]. The best-known causes are as follows: insufficient vascularization of the neotubule; the tension produced by mechanical traction exerted by the tissues on the anastomosis, and the packaging of the anastomosis itself, with the choice between the mechanical stapler or threads [3], [4]. Less important, but still influential, are factors related to the patient's health status such as age, BMI, and comorbidities such as diabetes or vascular diseases. Other minimally invasive endoscopic treatments for dehiscence include endoscopic clipping and placement of coated endoluminal protheses. Eso-SPONGE® has provided better results in less time than previous methods [5]. The device is positioned through endoscopic control under general anesthesia. After visualization of the dehiscence, the overtube is placed, which acts as a guide for insertion of the pre-shaped polyurethane sponge that is designed for connection to the suction system at −125 mmHg. The control is contextual and endoscopic. Repositioning takes place every 3 days [6], [7].

This work has been reported in line with the SCARE 2020 criteria [8] and it is compliant with the PROCESS Guidelines [9].

## 2 Case presentation

A 72-years-old Caucasian male with a history of atrial fibrillation, arterial hypertension came to our Department. He referred to have been a heavy smoker for over 40 years, no drugs abuse has been declared. His genetic and familiar history are negative for significant pathologies. He underwent a middle lobectomy in our center in 2011 for lung cancer. The patient returned to our attention for the detection of two synchronous neoplasms: a moderately differentiated adenocarcinoma of the esophagus (pT1b N0) and a pulmonary adenocarcinoma (pT3 N0). In December 2020, after neoadjuvant radio and chemotherapy, the patient underwent subtotal esophagectomy *sec*. Ivor Lewis with intrathoracic anastomosis and right lower lobectomy in posterolateral thoracotomy. In February 2021, he returned to our Unit due to the worsening of his general clinical conditions.

**Fig. 1:**
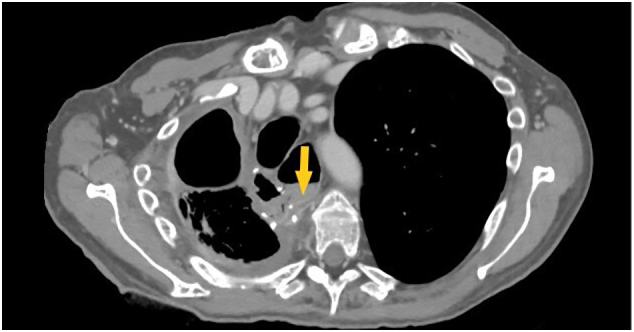
Postoperative CT scan showing leak of CE.

The patient followed a diagnostic check with a chest X-ray and then a chest-abdomen CT with contrast (C), which led to the evidence of anastomotic dehiscence with abundant spreading of food material into the pleural space (Fig. 1). A chest tube was placed to stabilize the patient's clinical condition. Antibiotic therapy was set to treat the pleural infection, and abundant daily pleural washes with saline solution were performed for about 20 days. After the infection had resolved, a minimally invasive treatment with the placement of an Eso-SPONGE® was used.

**Fig. 2:**
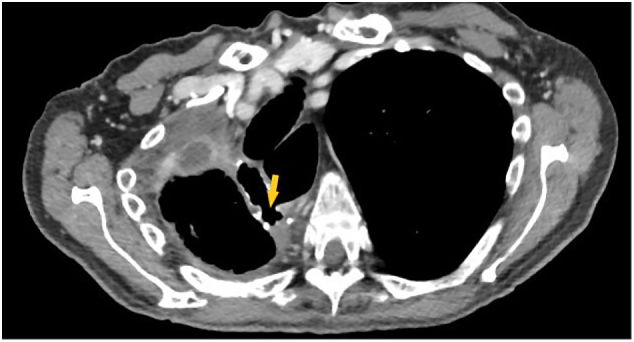
CT scan after the treatment with Eso-SPONGE®.

**Fig. 3:**
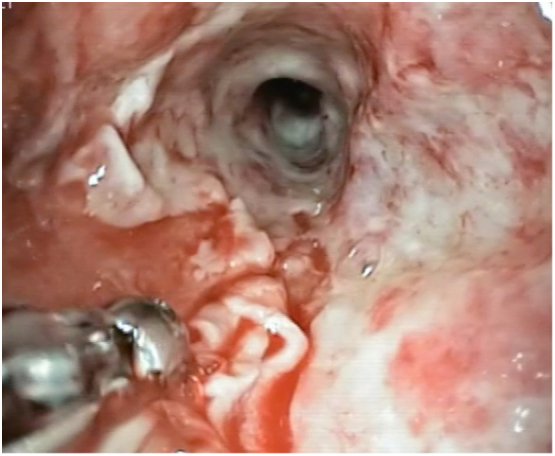
Curettage of the partial dehiscence.

**Fig. 4:**
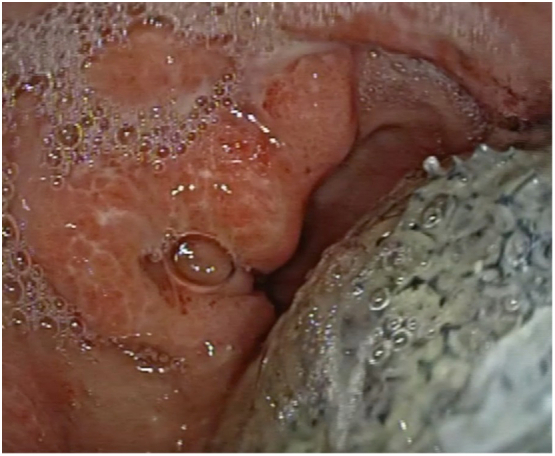
Eso-SPONGE® positioned into the partial dehiscence.

The positioning procedure of the device is as follows. The patient undergoes general anesthesia with endotracheal intubation. The nose-gastric tube is removed, and an exploratory esophagogastroduodenoscopy (EGDS) is performed to verify the level and diameter of dehiscence. Simultaneously, with the help of a biopsy clamp, a curettage of the area around the fistula and of the neoformed cavity is performed to revitalize the tissues and to remove the fibrin (Fig. 3). The endoscope is covered with a rubber over-tube that is placed under video control at the entrance of the dehiscence. Once the endoscope is extracted, the Eso-SPONGE® (already connected to the nose-gastric tube) is pushed through the over-tube in the correct position, and after the endoscopic check, it is connected to the suction VAC therapy at −125 mmHg. The sponge is replaced every 3 days (Fig. 4). The newly formed cavity from the dehiscence to the second EGDS, performed to remove and reposition the sponge, appeared with abundant tissue granulation. The procedure was well tolerated by the patient. After seven procedures, a neoformed wall was created, with complete channeling. The patient underwent the necessary sealing tests by gastrographin and a chest-abdomen CT scan with contrast to confirm a good lumen of the esophagus and the absence of spreading of Cin the pleural cavity (Fig. 2). This allowed us to reintroduce nutrients to the patient, which consisted of a water-based diet firstly and then a creamy diet, with very satisfying results. After three months the patient showed no signs of dysphagia, regurgitation, or difficulty in digestion, while feeding on a free diet.

## 3 Clinical discussion

Anastomotic dehiscence is a complication that can occur with up to 30% frequency and, in unstable patients, can lead to death. In acute, non-stabilized patients, treatments are re-surgery, endoscopic clipping, or the use of glues, all of which have the sole purpose of stabilizing the clinical conditions of the patient to avoid exitus. If the clinical conditions of the patient are led to stability and it becomes chronic, the most used method to date for the treatment of anastomotic dehiscence is endoscopic stenting with a waterproof-coated prothesis. However, recent evidence has shown that Eso-SPONGE® has a higher success rate than a stenting procedure (86.4% vs. 60.9%) and lower treatment times (26.5 days vs. 36) [10], [11]. The costs of Eso-SPONGE® are higher than those of stenting, but are balanced by the reduction of the days of hospitalization and justified by the best progress in the healing of patients [12]. The critical aspects of the procedure are related to the management of the spaces in the oral cavity of the patient and to the protection of the airways, as for a possible conflict which may be created between the endotracheal tube and the over-tube, both of large caliber.

## 4 Conclusion

Although we are fully conscious that this is not the first case of the use of Eso-sponge, our clinical case would like to take the attention as the first placement of this procedure in our center in difficult clinical conditions. In fact the patient showed two different cancers, one at the right lower lobe and one at the level of the low esophagus with serious comorbidities.

This means that this procedure is useful in complex cases even during the first procedures and in case of a long-term dehiscence.

In chronic patients, the use of Eso-SPONGE® in the treatment of anastomotic dehiscence has proved to be a very effective method with even better results than the already existing methods. In addition, the repeatability and the ease of positioning makes it accessible even in suburban centers, provided they have a specialized digestive endoscopy.

## Ethical approval

None.

## Provenance and peer review

Not commissioned, externally peer-reviewed.

## Sources of funding

No founding.

## Consent

Written informed consent was obtained from the patient for publication of this case report and accompanying images. A copy of the written consent is available for review by the Editor-in-Chief of this journal on request.

## Research registration (for case reports detailing a new surgical technique or new equipment/technology)

NA.

## Guarantor

Beatrice Aramini MD PhD, Prof. Franco Stella MD PhD.

## Declaration of competing interest

No conflict of interest.
